# Stress-Hormone Dynamics and Working Memory in Healthy Women Who Use Oral Contraceptives *Versus* Non-Users

**DOI:** 10.3389/fendo.2021.731994

**Published:** 2021-11-08

**Authors:** Emma Sofie Høgsted, Camilla Borgsted, Vibeke H. Dam, Arafat Nasser, Niklas Rye Jørgensen, Brice Ozenne, Dea Siggaard Stenbæk, Vibe G. Frokjaer

**Affiliations:** ^1^ Neurobiology Research Unit, Copenhagen University Hospital–Rigshospitalet, Copenhagen, Denmark; ^2^ Mental Health Services, Capital Region of Denmark, Copenhagen, Denmark; ^3^ Department of Clinical Medicine, University of Copenhagen, Copenhagen, Denmark; ^4^ Department of Clinical Biochemistry Centre of Diagnostic Investigations, Copenhagen University Hospital–Rigshospitalet, Copenhagen, Denmark; ^5^ Department of Public Health, Section of Biostatistics, University of Copenhagen, Copenhagen, Denmark; ^6^ Department of Psychology, Faculty of Social Sciences, University of Copenhagen, Copenhagen, Denmark

**Keywords:** cortisol, oral contraceptives, hormonal contraceptives, working memory, estradiol, depression, cortisol awakening response, HPA-axis

## Abstract

**Background:**

Women who use oral contraceptives (OCs) may have a higher risk of developing a depression, which is associated with both vulnerability to stress and cognitive dysfunction. OCs disrupt the hypothalamic-pituitary-gonadal (HPG) axis by suppressing endogenous sex steroid production including estradiol. The HPG axis and the hypothalamic-pituitary-adrenal (HPA) axis are known to interact, possibly through modulations driven by estradiol. OCs may affect HPA regulation capacity, i.e., disturb cortisol dynamics such as the cortisol awakening response (CAR), and influence cognition such as working memory (WM). We hypothesize that OC use is associated with blunted cortisol dynamics and impaired WM performance relative to non-users.

**Methods:**

Data from 78 healthy women in the reproductive age were available from the CIMBI database. We evaluated if CAR and WM differed between OC users (n=25) and non-users (n=53) and if the level of estradiol modulated the OC use effect on CAR or WM in generalized least square models.

**Results:**

We found that OC users had a blunted CAR (p= 0.006) corresponding to a 61% reduction relative to non-users; however, no estradiol-BY-OC use interaction effect was observed on CAR. Also, OC users had higher cortisol levels at awakening compared to non-users (p = 0.03). We observed no effect of OC use or an estradiol-BY-OC use interaction effect on WM. Also, within the OC user group, neither CAR nor WM was associated with suppressed estradiol. CAR was not associated with WM.

**Conclusion:**

Healthy women who use OCs have blunted cortisol dynamics relative to non-users. However, we could not detect OC use effects on working memory in our sample size. We speculate that disrupted cortisol dynamics may be important for the emergence of depressive symptoms in OC users.

## Introduction

Combined synthetic estrogen and progestogen OCs are widely used by women in the reproductive age. More than 50% of Scandinavian women begin using hormonal contraceptives before the age of 17 (1), and 42% of Danish women of fertile age use OCs (2). Women who start treatment with oral contraceptives (OCs) are at a higher risk for developing a depressive episode relative to non-users (3). This phenomenon is particularly pronounced in adolescence (3), as also supported by recent independent findings showing that adolescents who use OCs report more depressive symptoms (4) and are more likely to start psychotropic drugs (5) compared to their unmedicated peers. Also, adolescent girls who use OCs have long-lasting vulnerability for depression into adulthood even after discontinuing OCs (6). Depressive episodes are associated with both vulnerability to stress and cognitive dysfunction (7).

The hypothalamic-pituitary-adrenal (HPA) axis dysfunction is a key feature of MDD and several other psychiatric diseases (8). The HPA axis generates diurnal cortisol rhythms and responses to stress or other stimuli. A dynamic cortisol response is considered critical for a healthy adaptation to stress and therefore support resilience (9). Regulation of the HPA axis has suggested to be affected by OCs (10) as blunted or absent cortisol responses to psychosocial stress tests have been observed in healthy women using OCs (10–13). Thus, OCs may, at least in some women, add to the risk of developing psychiatric disorders by increasing vulnerability to psychosocial stress. OCs disrupt the Hypothalamic-Pituitary-Gonadal (HPG) axis and consequently suppress endogenous ovarian hormone production, including estradiol (14). The HPG and HPA axes are intimately related, and sex steroid receptors are pervasively expressed in key parts of the neural circuitry controlling the HPA axis (15). Overall, estradiol plays a significant role in HPG and HPA axis interactions (15). Thus, OC-induced suppression of the HPG axis including estradiol-suppression may well affect the HPA axis and thereby cortisol dynamics, which is putatively critical to mental health. So far, the leading explanation for the observed changes in cortisol dynamics in OC users has been that cortisol binding globulin (CBG) increases in response to OCs and thus less free cortisol is available (13, 16, 17). However, as suggested by Kirschbaum et al. (17), HPA and HPG relations might also play a role.

One way to characterize the HPA axis is by examining the cortisol awakening response (CAR), which is a superimposition on the circadian rhythm of cortisol release that occurs in response to awakening (18). CAR can be easily assessed by home-sampling of saliva, allowing the observation of HPA dynamics in a natural setting. In contrast to more extreme stress test responses, CAR reflects the HPA axis output in a basic everyday condition that may be particularly relevant to mental health risk and resilience mechanism associated with OC use. CAR is known to be influenced by various state and trait factors (19). So far, cortisol responses in OC users have mostly been examined in relation to high intensity HPA axis stimuli such as psychosocial stress or pain (11–13, 20–22), whereas data on OC effects on diurnal features of the HPA axis such as CAR, e.g., responses to an every-day HPA axis stimuli such as awakening, are sparse and inconclusive. Also, early studies did not include factors later known to affect CAR (17–19, 23), and one study only examined adolescent OC users without providing full CAR characterization (13). In this study we include several factors known to affect CAR as suggested by Stalder et al. (2010) (18) and provide a full CAR characterization.

We further want to examine the relationship between WM and OC use as sex steroids, especially estradiol, appear to affect cognitive functions and in particular working memory (WM) (24), which notably is also impaired in MDD (25). WM is the ability to temporarily store and manipulate information that is required to carry out cognitive tasks such as comprehension, thinking and reasoning. In healthy naturally cycling women, WM seems to be impaired during menstrual bleeding when estradiol is low, and in pregnant women WM is improved in late pregnancy when estradiol is high (26, 27). Overall some studies demonstrated better performance by OC users on some cognitive domains by such as emotional memory, susceptibility to false memories and especially verbal memory, while others found no difference in cognition examining visuo-spatial memory, verbal fluency, and attention (28, 29).

We hypothesize that women who use OC relative to non-users have a blunted CAR, which may be associated with low endogenous levels of estradiol. We further explore if OC use is associated with worse performance on cognitive tests of WM, possibly in a manner dependent on estradiol.

## Materials and Methods

### Participants

**Figure 1:**
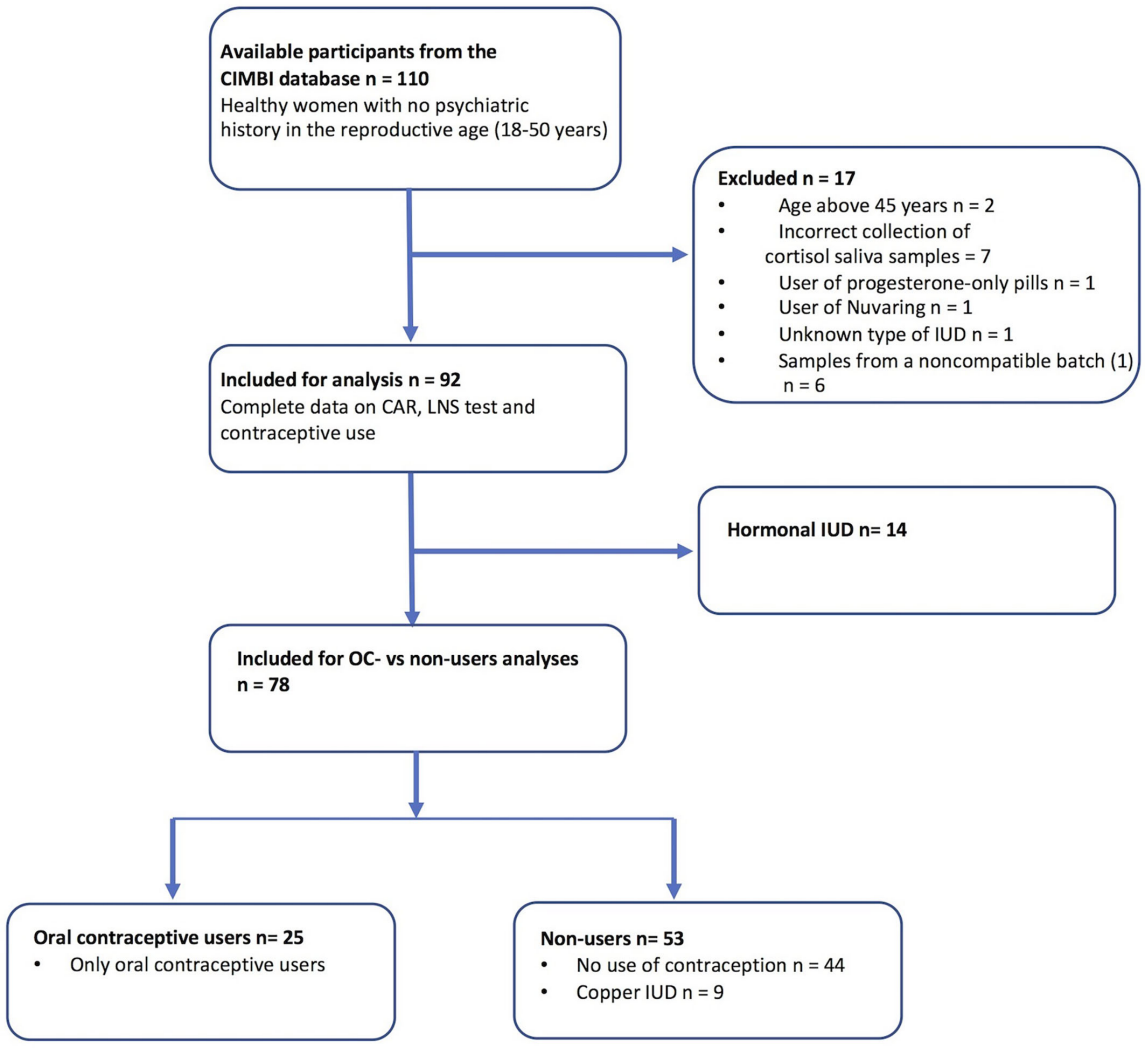
Overview on study population selection from the CIMBI database. Participants recruited from the Center for Integrated Molecular Brain Imaging (CIMBI) database. Inclusion criteria were healthy women with no psychiatric history in the reproductive age <50 years. A complete dataset was required with concurrent information on cortisol awakening response (CAR), Letter-Number-Sequence (LNS) test, and contraceptive use. Exclusion criteria were (1) women above 45 years of age with early menopause or unknown menopausal status measured with follicle stimulation hormone (FSH), (2) incorrect collection of home cortisol samples, and (3) hormonal contraceptives other than OCs or intrauterine devices (IUDs). Further, six women had incomparable cortisol data due to batch variations of the cortisol saliva samples.

Data were available from the Center for Integrated Molecular Brain Imaging (CIMBI) database (30). We included data from healthy women within the reproductive age between 18 and 50 years of age with no psychiatric history. All the women had concurrent information on contraceptive use, cortisol dynamics (CAR), and cognitive functioning (WM). Overview on study population selection from the CIMBI database is shown in **Figure 1**. The CIMBI database contained data from 110 healthy women that met the inclusion criteria. Our exclusion criteria were (1) Women above 45 with early menopause or unknown menopausal status measured with follicle stimulating hormone, (2) incorrect collection of home cortisol samples, and (3) hormonal contraceptives other than OCs or hormonal intrauterine devices (IUDs). Eighteen women were excluded from the study: two women >45 years of age had missing follicle stimulating hormone data, thus menopausal status was unknown, and seven women were excluded due to non-compliance when collecting home cortisol saliva samples. Further, we excluded a user of progestin-only pills, a nuva-ring user, and one woman with unspecified type of IUD. Cortisol saliva samples were analyzed in larger batches to minimize experimental noise. One small (n= 6) batch of saliva samples could not be efficiently compared to later batches as they significantly differed from internal standard quality control samples, which are run routinely in each batch; consequently, we excluded six women with incomparable saliva samples. Thus, data from 78 women were available for analysis. Fifty-three women did not use hormonal contraception (non-users): 44 women reported no use of contraceptives and nine women had copper IUDs (see **Figure 1**). Fourteen women using hormonal IUDs were included for additional analysis but were not included in the OC user group or non-user group (see **Figure 1** for overview on study population selection). All OC users used combined ethinylestradiol and progesterone contraception, however with slightly different types of gestagene and small variations in estradiol dose. None of the participants had a history of psychiatric or current severe somatic illness. Participants from the database had been recruited for different neuroimaging projects between 2007 and 2018, and all women provided written consent for inclusion in the CIMBI database. All projects were approved by the Ethics Committee of Copenhagen and Frederiksberg or of Region, Denmark [(KF)01-274821, (KF)01-2006-20, H-15004506, H-1-2010-085, H-4-2012-105, H-6-2014-057, H-15017713].

### Clinical and Genetic Measures

All participants were screened with physical and neurological examinations and clinical blood samples. Information on the serotonin transporter genotype (5-HTTLPR) was obtained in terms of 5-HTTLPR high-expressing (LA/LA) or low-expressing (LG or S carrier) variants (31). This enabled us to test if those variants were associated with CAR, as earlier studies have indicated associations with cortisol responses to a standardized psychosocial stress exposure (31). Information on smoking and daily number of cigarettes was further collected. Plasma estradiol samples were obtained maximum 3 days after the collection of the saliva cortisol samples. Twenty-four participants had their estradiol blood samples collected later than 3 days from saliva collection or WM test. These women were excluded from analyses. Thus, estradiol data were available for analysis on 49 women for CAR analysis (OC users: 15; non-users: 34) and 52 for WM analysis (OC users: 14; Non-users: 38) out of the 78 women (see Figure 6 in supplementary for selection process). The menstrual phase for the non-user women was unknown, except for 26 out of 53 non-user women who had their blood samples collected during the follicular phase as defined by the design of the study they participated in. Hospital analysis method of plasma sex steroid levels was based on antibody reagents from Estradiol II and Elecsys^®^ Estradiol III, Roche. Comparability of estradiol analysis is treated in further details in Larsen et al., 2020. Seventeen out of 73 estradiol samples were below the lower detection limit (0.04 and 0.09 nmol/L, respectively).

### Cortisol Measurements

Saliva samples were collected in Salivette® tubes (Sarstedt, Neubringen, Germany). We measured the cortisol awakening response (CAR), i.e., the dynamic increase in cortisol levels that occurs within the first hour upon morning awakening. For the assessment of CAR, participants were instructed to collect saliva sample immediately after awakening and after 15, 30, 45, and 60 min by chewing a swab until it was fully saturated with saliva. Participants were told to avoid food, drinking, brushing teeth, and smoking during the first hour after waking up. Further, the participants were instructed to take a saliva sample at bedtime the same day as collection of CAR. The women noted whether the CAR samples were collected on a work, study, or rest day and at which time the samples were collected including time at awakening. Our inclusion criteria assured that when saliva samples were collected more than 10 min after awakening, the datasets were not included in our analyses. The saliva samples collected at home were stored in the refrigerator and returned to the laboratory the day after completion of sampling, and if collected during the weekend, maximum 3 days after collection. The CAR measurements were computed as the area under the curve with respect to increase from baseline at awakening (AUCi). The AUCi captures change over multiple time points and is thereby an index of change (32). The saliva samples were analyzed in different batches across the collection period (2007 to 2018) to minimize experimental noise. We present data from six different batches. Participant training, instructions, home-sampling procedures, storing, and cortisol analyses were carried out as described in Frokjaer et al., 2013. Salivary cortisol concentrations were determined by an electrochemiluminescence immunoassay (ECLIA) method on Modular Analytics E170 equipment (Roche, Mannheim, Germany) and for two of the most recent batches by a chemiluminescence immunoassay (CLIA) method on the IDS-iSYS automatic analyzer (IDS PLC, Boldon, UK). The two methods were equally distributed in the two groups. The intra- and inter-assay variation of both methods was <15%.

### Cognitive Tests

All cognitive tests were performed by or supervised by a trained neuropsychologist. The single tests in the program are described in further details in Dam et al., 2020. The main task used to index WM function was the Letter Number Sequencing (LNS) test from WAIS-III. The LNS is a verbal WM task where the test subject is asked to listen to a jumbled sequence of letters and numbers before mentally sorting and reciting them back starting with the numbers in numerical order followed by the letters in alphabetical order. Number of completed trials is the main outcome of the test with scores ranging from 0 to 23. As a second test we used the Symbol Digit Modalities Test (SDMT), which provides a measure of psychomotor speed and WM capacity. The SDMT involves visual processing and is a paper-and-pencil test of 90 seconds, in which the test subject has to translate abstract shapes to numbers based on a translation key that the test subject must learn and hold in working memory in order to increase speed. Number of correctly translated symbols is the main outcome of the test. Data on SDMT were missing for two participants. For descriptive purposes, intelligence factor (IQ) was tested with Reynolds Intellectual Screening Test (RIST), which provides a proxy measure of the general, age-adjusted IQ. The time interval between cortisol saliva samples and WM tests was 0 to 112 days with a mean of 3 days. Seventy-one out of 78 participants had less than 2 weeks between WM test and cortisol saliva sample collection.

### Questionnaires

The participants’ mental well-being was assessed with Cohen’s Perceived Stress test (Cohen’s PSS), Major Depression Inventory (MDI), and Profile of Mood States (POMS). PSS was placed at the day of home collection of the cortisol saliva samples. MDI indexes symptoms of depression according to the ICD-10 diagnostic system and can be used as a screening tool for MDD. The individual subscales of the POMS can be summarized in the Total Mood Disturbances (TMD). Additionally, information on education and sleep quality was collected with questionnaires. Education level was scored on a five-point Likert scale: One corresponded to having no vocational degree and five if they had more than four years of higher academic education. Data on education level were missing for three participants. Information on sleep quality was obtained with a global score from Pittsburgh Sleep Quality Index (PSQI) with higher scores indicating worse sleep. PSQI was completed 0–5 days before cortisol sample collection. PSQI questionnaire data were available for 66 women (OC users: 21; Non-user: 45).

### Statistics

We used a Welch’s t-test for continuous measures and a Fisher’s test for categorical parameters to determine if there were any group differences in demographics, cognition, psychometrics, and hormone levels. Our main analysis was the association between AUCi CAR and OC use. As secondary cortisol analyses, we examined (1) differences in cortisol at wake-up (wake-up cortisol) and evening cortisol values between OC users and non-users. (2) In a subgroup of 49 women (OC user: 15; non-user: 34), we investigated if there was an estradiol-BY-OC use interaction effect on CAR. (3) We further tested an association between CAR and hormonal IUD (IUD-users: n=14; non-users: n=53). All CAR and cortisol analyses were tested in generalized least squares models and adjusted for age, BMI (which differed between OC users and non-users), and work day status (work/study day *vs.* rest day) as, in the earlier studies, anticipation of a working day has been shown to be associated with an enhanced CAR (18).

Follow-up sensitivity analyses were performed to evaluate the robustness of identified significant associations. Alternative models were tested by adjusting for relevant covariates that could potentially affect CAR or the HPA axis according to guidelines (18). We specifically considered smoking and 5-HTTLPR genotype (LA/LA *versus* not-LA/LA) as some studies suggest that they may affect the HPA axis (31, 33, 34). We further examined state covariates proposed by consensus guidelines for the assessment of CAR (18): season (summer *vs.* winter), BMI, TMD, PSQI global sleep quality index, and work day status (work/study day *vs.* rest day) (see **Table 2**). Even though we already tested the effect of work day *vs.* rest day on CAR, we further tested if time at awakening could influence our main model by replacing work up status with awakening time (reported in supplementary material only). To test if absolute cortisol values at wake-up drove a group difference in AUCi CAR, we also evaluated the effect of adjusting our main analysis for cortisol awakening values. As a second sensitivity analysis, we used the generalized least squares test to account for possible batch effects. We thus accounted for potential random effects in each batch due to variations in technical analyses.

In our main working memory analyses, a generalized least squares test was performed to test for an effect of OC use on working memory [LNS (n=75)/SDMT (n=73)] (see **Figure 7** in supplementary for selection of population for WM analysis). As secondary working memory analyses, we investigated (1) if there was an estradiol-BY-OC use interaction effect on WM (SDMT/LNS) in a subgroup of 52 women (OC user: 15; non-user: 38) (see **Figure 6** in supplementary for selection of population for estradiol analysis). (2) Within the OC user group we examined if plasma estradiol was associated with CAR (n = 15) or WM (n=14). (3) Finally, we tested if a relationship between WM and CAR existed in a generalized least squares test in the manuscript adjusted for age. The analyses were constrained to data points that were collected maximum 14 days apart. All WM models were adjusted for age and education score except the analysis within the OC user group as they all had identical education scores. Since WM is a component of IQ scoring, we chose to adjust for education score rather than IQ (35). See supplementary material for regression report on WM and OC use association adjusted for parameters differing between the OC user and non-user group. We did not use outlier detection or incorporate outlier exclusion in our analyses. All statistical tests and graphical presentations were performed in R Statistics version 1.2.5001 (R Core Team (2017). R: A language and environment for statistical computing. R Foundation for Statistical Computing, Vienna, Austria. URL (https://www.R-project.org/).

## Results

### Participants

Demographic, psychometric, cognitive, and endogenous hormonal data of the study population are presented in **Table 1**. The two groups (OC users *vs.* non-users) were similar in mean age, IQ, education score, depressive symptoms (MDI), stress (PSS), and distribution of 5-HTTLPR-genotypes. The groups differed on several parameters even though they all were within the normal range. On sleep quality (PSQI global score), the OC users displayed worse sleep within the last month compared to non-users with a mean above the cutoff score of 5, indicating impaired sleep (p-value=0.09). OC users had worse scores on mood (TMD) compared to non-users (p-value = 0.05). Both groups were clearly within the mentally healthy spectrum as also supported by very low MDI scores. OC users scored higher on education level (p-value= 0.02) and in the SDMT-test (p-value=0.06). As expected, compared to the non-users, OC users had suppressed endogenous estradiol and progesterone levels (estradiol p-value=0.07, progesterone p-value=0.01). OC users had progesterone levels below 5 nmol/L, suggesting anovulation and good compliance to oral contraceptives. Data on estradiol levels were missing for five women, and data on progesterone levels were missing for six women. Most women were within the normal range of BMI (weight in kg/height in m^2^), except three who were underweight (BMI <18,5), 11 who were overweight (25–30), and one who was obese (BMI >30). BMI was significantly higher in non-users. The frequencies of work day status (p-value=0.01) and BMI (p-value=0.09) were distributed differently, but the frequencies of 5HTLLPR genotype and smoking were not (p-value>0.39). Most participants reported no use of other medications, two women used mild non-steroid allergy medication in terms of antihistamines, and one of these also had a nasal steroid spray prescribed although she did not use it.

**Table 1:** Demographic, cognitive, psychometric, and hormonal data.

Clinical parameters	OC-user (n = 25)	Non-user (n = 53)	Range	p-values	n
Age	23.6 (2.42)	25.1 (5.21)	18–39	0.09	78
BMI	21.5 (1.7)	23.2 (2.8)	17–32	0.002	78
IQ	109 (6.8)	110 (7.26)	96–126	0.53	78
Education score	4.7 (0.8)	4.1 (1.5)	1–5	0.03	75
LNS	12.5 (2.6)	12.6 (2.6)	6–19	0.94	78
SDMT	68.2 (9.8)	68.2 (10)	44–87	0.06	76
Cohen’s PSS	7.0 (5.5)	8.2 (6.4)	0–23	0.39	78
MDI	5.1 (2.9)	5.1 (3.4)	0–15	0.93	76
TMD	-4.2 (11.8)	2.4 (16.1)	–21–58	0.05	76
Sleep quality	5.1 (2.9)	3.8 (2.1)	1–10	0.09	66
P-Estradiol nmol/L	0.12 (0.2)	0.48 (1.4)	0.04–10	0.07	74
P-Progesterone nmol/L	0.99 (0.43)	3.93 (7.74)	0.4–41	0.01	71
**Categorical variables**	**OC-user (n = 25)**	**Non-user (n = 53)**		**p-values**	
Smoking					
- Non-smokers	96% (n = 24)	83% (n = 44)		0.39
- Light smokers	4% (n = 1)	7% (n = 4)		
- Intermediate smokers	0% (n = 0)	4% (n = 2)		
- Missing value	0% (n = 0)	6% (n = 3)		
5-HTLLPR genotype					
- LA/LA	28% (n = 7)	23% (n = 12)		0.78
- Other genotypes	72% (n = 18)	77% (n = 41)		
Day of cortisol saliva samples					
- Work/study day	36% (n = 9)	68% (n = 36)		0.01
- Rest day	64% (n = 16)	32% (n = 17)		
BMI					
- Underweight (<18)	4% (n = 1)	4% (n = 2)		0.09
- Normal weight (18–25)	92% (n = 23)	75% (n = 40)		
- Overweight	4% (n = 1)	19% (n = 10)		
- Obese	0% (n = 0)	2% (n = 1)		

Mean, standard deviation, and range are shown for clinical parameters in each group. The categorical variables are presented showing the distribution of smoking, 5-HTLLPR genotype, whether the cortisol saliva samples were collected on a work, study, or rest day, and BMI. For clinical parameters, statistical differences were calculated with Welch’s t-test, and for the categorial variables, differences were calculated with Fisher’s test. Sleep quality was assessed with Pittsburgh Sleep Quality Index (PSQI). PSQI global score ranges overall sleep quality from 0 to 21 with higher scores indicating worse sleep. Total mood disturbance (TMD) ranging from 0 to 200 with higher scores indicating mood disturbances. Body mass index (BMI), Letter-Number-Sequence test (LNS), Cohen’s Perceived Stress test (Cohen’s PSS), Major Depression Inventory (MDI). Light smoker = max 5 cigarettes per day, intermediate smoker = 5–15 cigarettes per day. *For calculation of Fischer’s test, we pooled BMI under 25 versus BMI above 25.

### Association Between CAR and OC Use

**Figure 2:**
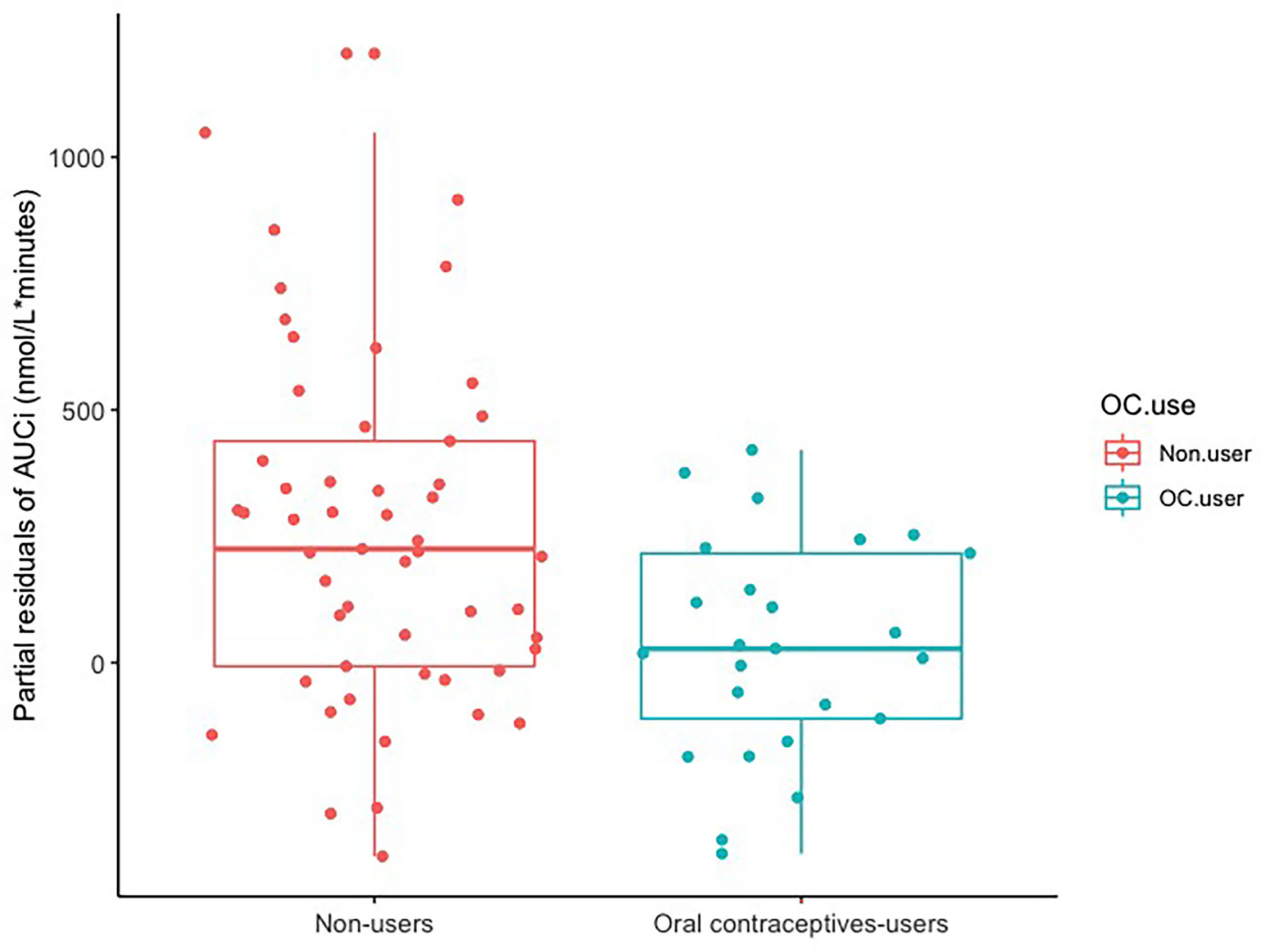
CAR in oral contraceptive users *versus* non-users. Boxplot showing partial residuals of CAR AUCi (nmol/L*minutes) to remove the effect of age, BMI and work day status (work/study day *vs.* rest day). Oral contraceptive users display a significantly reduced AUCi CAR compared to non-users (p-value= 0.006). CAR, cortisol awakening response; AUCi, area under the curve with respects to increase.

**Figure 3:**
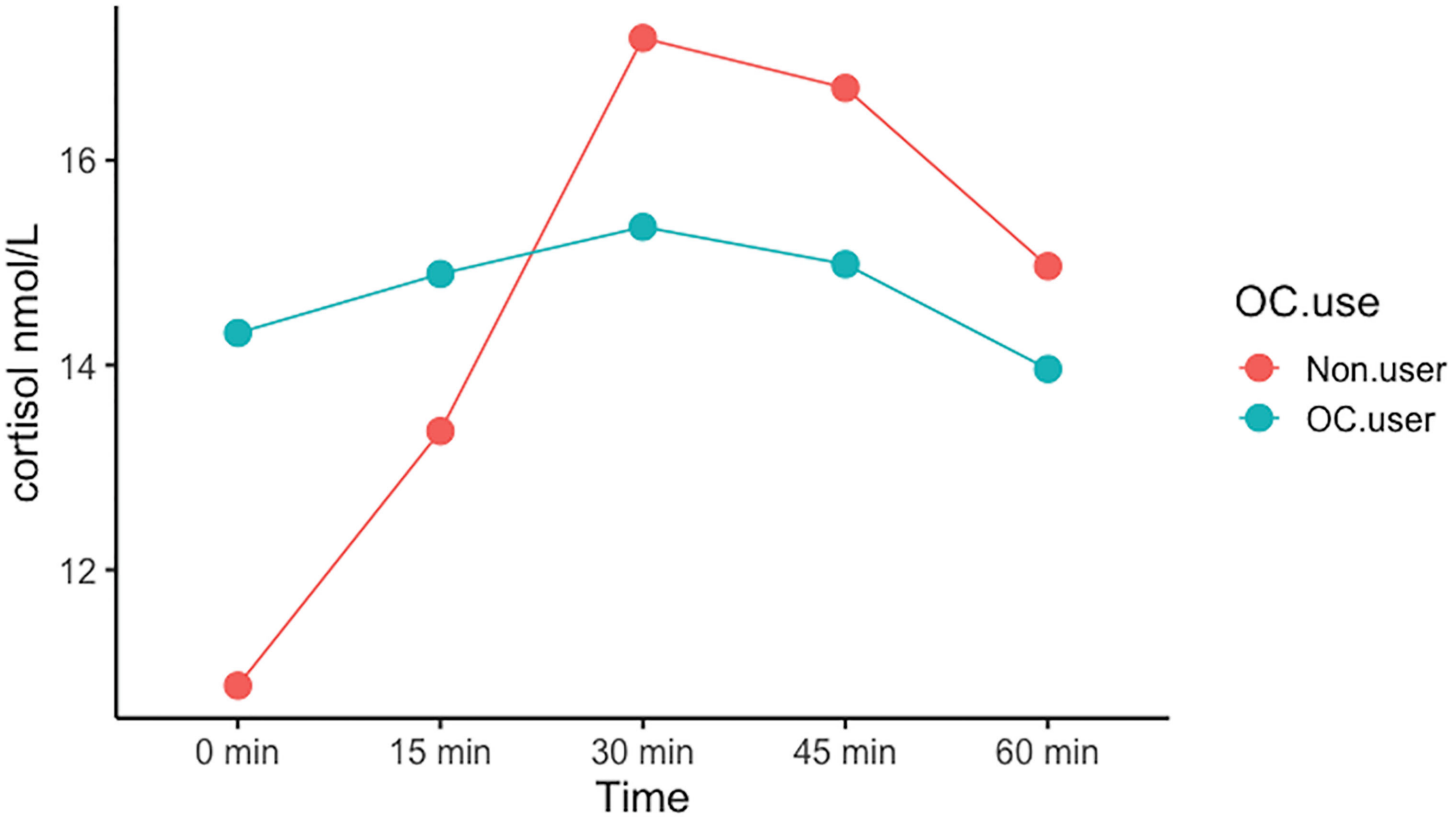
The cortisol awakening response in OC-users and non-users. Mean cortisol values at each time point during the first hour after wake up (0 min) depicting the cortisol awakening response. The 95% confident intervals are presented as the shadowed area surrounding the line.

OC users displayed a significantly diminished AUCi CAR by β= −203 nmol/L*minutes (CI: [−343; −63], p-value= 0.006) relative to non-users, corresponding to a 61% reduction (see **Figure 2** and **Table 2**: model B) in a model adjusted for age, work day status, and BMI. This indicates a blunted CAR in OC users, as illustrated in **Figure 2**. In our secondary analyses, (1) we observed no estradiol-BY-OC use-effect on CAR (β= −564, CI 95% [−3,075;1,946], p-value= 0.66). (2) Within the OC user group, estradiol was not associated with CAR (β= −32, CI 95% [−100;35], p-value= 0.34). Also, OC users had higher cortisol levels at awakening compared to non-users (β= 3.43, CI 95% [0.41;6.46], p-value= 0.03), but absolute evening cortisol did not differ (β= −0.22, CI 95% [−1.34;9.14], p-value= 0.25). See **Figure 3** for unadjusted mean values for each CAR time point in the two groups. (3) Effects from hormonal IUD relative to naturally cycling non-users were not evident on CAR (β= 122, CI 95% [−83;328], p-value= 0.25). When including the hormonal IUD group in the non-user group, the results were largely similar to the results above (when constraining our analyses to compare OC users with natural cycling women) (see supplementary material).

**Table 2:** The effect of OC-use on CAR evaluated in generalized least square analyses in alternative models with increasing complexity.

Covariates for adjustment	Effect (nmol/L*minutes)	95% CI	p-value
A. No adjustment	−228	[−354; −103]	<0.001
B. Age, work day status, BMI	−203	[−343; −63]	0.006
C. Age, BMI, Cohen’s PSS, 5HTTLPR genotype, TMD, smoking, work day status, season, sleep quality*	−238	[−418; −57]	0.013

Model B was chosen as our main model. Work day status describes whether cortisol saliva samples were collected on a work/study or rest day. 5-HTTLPR genotype status is defined as LA/LA versus not-LA/LA. Sleep quality was assessed with global score of the Pittsburgh Sleep Quality Index (PSQI). Season, summer versus winter. Cohen’s PSS, Cohen’s Perceived stress scale; TMD, total mood disturbance. * Model C only includes 63 participants due to missing PSQI data (n=12) and missing TMD data (n=2).

Follow-up sensitivity analysis showed that BMI contributed to the CAR model (**Table 2**). We further evaluated age, season (winter *vs.* summer), Cohen’s PSS, TMD, smoking, sleep quality, and 5-HTTLPR genotype as potential significant covariates for a CAR and OC use association. Those variables did not contribute to the reported results (**Table 2**), nor were they correlated with the CAR (p-value> 0.35). As expected, CAR was moderately intercorrelated with absolute cortisol levels at awakening (Pearson correlation = −0.45). Nevertheless, when adjusting our main model for awakening cortisol levels, CAR remained to be associated with OC use at a borderline significant level (β:−122, CI 95% [−245;−2.25], p-value = 0.058).

**Figure 4:**
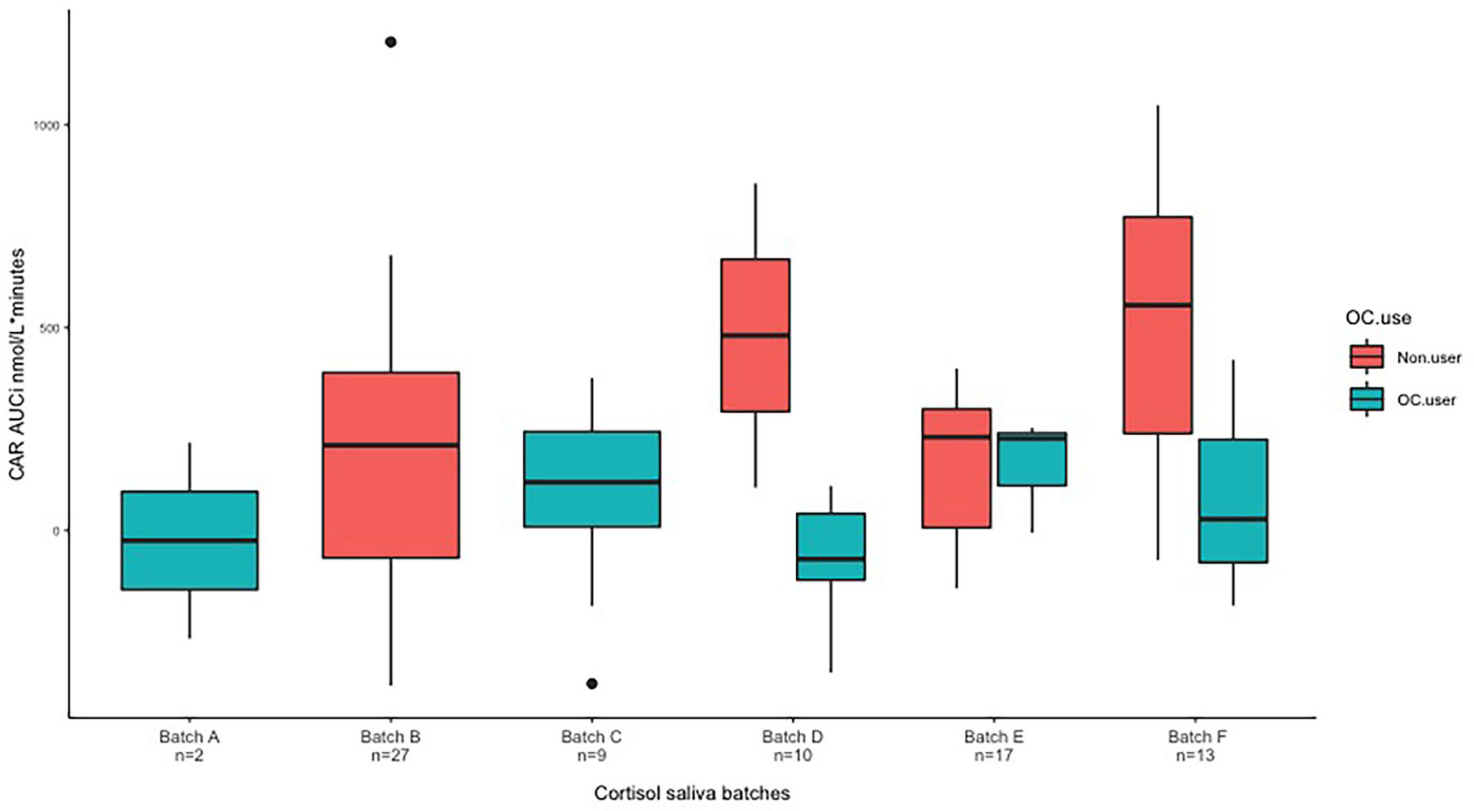
Variance seen by boxplot based on unadjusted observations. We here present data from six different batches. Batch D: non-users n = 2, OC-users n = 8. Batch E: Non-users n = 14, OC-users n = 3. Batch F: non-users n = 10, OC-users n = 3. CAR, cortisol awakening response; AUCi, area under the curve with respect to increase.

Cortisol data from six different batches were pooled in the present study (see **Figure 4**). We evaluated possible batch effects by adding to the main CAR model an additive batch effect on the mean and a multiplicative batch effect on the variance. The resulting model was fitted using generalized least square. The difference in batch effect size compared to effects in the main CAR analysis was 16% (Batch analysis β: −260 and CAR analysis β: −203) with similar p-values (p-value=0.007 and p-value=0.006). We saw no evidence for substantial batch effects. Therefore, pooling of the six batches did not drive the outcome of our main analysis.

### OC Use and Working Memory

**Figure 5:**
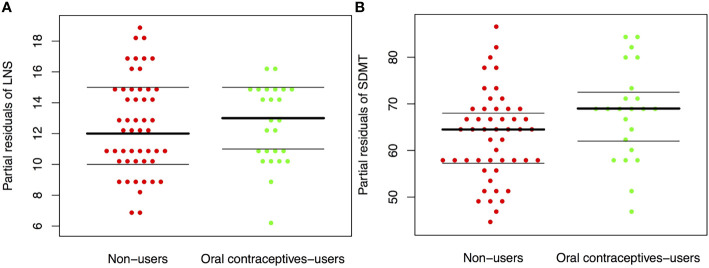
**(A, B)** Working memory in oral contraceptive users *versus* non-users. Boxplot showing partial residuals of working memory test to remove the effect of age and education based on a generalized least square model. Working memory was tested with the Letter-Number-Sequencing (LNS) **(A)** test and the Symbol-Digit-Modalities-test (SDMT) **(B)** in oral contraceptive-users (OC-users) and women using no oral contraceptives (non-users). There was no difference between OC-users and non-users on the LNS (p = 0.9), but a trend towards better performance in OC-users was observed on the SDMT (p = 0.14). WM, working memory; LNS, Letter-number-sequence test; SDMT, Symbol-Digit-Modalities test; OC, oral contraceptive; CAR, cortisol awakening response.

In our main analyses we observed a trend towards better SDMT performance in OC users (β= 3.88, CI 95%: [−1.2; 8.96], p= 0.14). In contrast, we found no OC use effect on LNS (β = 0.09, CI 95% [−1.48; 1.3], p-value= 0.9) (see **Figures 5A, B**). In our secondary analyses, (1) we saw no estradiol-BY-OC use-effect on LNS or SDMT. (2) Likewise, in an analysis constrained to the OC user group only, estradiol was not associated with LNS or SDMT. (3) Finally, we found no evidence for an association between CAR and LNS or SDMT. All results from secondary analysis are presented in **Table 3**.

**Table 3:** Results from secondary working memory analyses.

Secondary WM analysis		Effect	95% CI	P-value	n
**(1) Estradiol-BY-OC-use interactions**					
	LNS	−4.61	[−27; 18]	0.68	51
	SDMT	0.32	[−78; 78]	0.99	51
**(2) Estradiol-WM associations within OC-users**					
	LNS	−4.7	[−29; 19]	0.70	15
	SDMT	1.6	[−79; 76]	0.97	15
**(3) CAR-WM associations**					
	LNS	0.0002	[−0.002; 0.002]	0.88	66
	SDMT	0.0002	[−0.007; 0.007]	0.96	66

WM, working memory; LNS, Letter-number-sequence test; SDMT, Symbol-Digit-Modalities-test; OC, oral contraceptive; CAR, cortisol awakening response.

## Discussion

We here report the effect of OC use on CAR and WM in a group of healthy premenopausal women. We observed that healthy women who use OCs have blunted HPA axis dynamics in terms of CAR with a 61% reduction, compared to non-users, and at the same time higher absolute levels of cortisol at awakening. Meanwhile, we observed no statistically significant association between OC use and WM, except for a trend towards better visual WM performance in OC users. Furthermore, no group-dependent effects were observed in the association between estradiol and CAR or WM.

### OC Use and HPA Dynamics

As hypothesized, we found that OC users displayed a significantly diminished CAR with a 61% reduction relative to non-users (**Figure 2** and **Table 2**: model B) in a model adjusted for age, work day status, and BMI, consistent with a blunted CAR in OC users, a finding that was robust also when tested in alternative models. Our findings align with prior studies examining CAR and cortisol response to a social stress test in OC users *versus* non-users, which have reported attenuated cortisol responses in OC users (11–13, 36). Taken together with previous studies, our finding indicates that OC users display blunted HPA axis dynamics. Earlier attempts to address CAR differences between OC users and non-users have been inconclusive as they did not adjust for factors later known to clearly affect CAR, such as work day *vs.* rest day, smoking, and sleep quality (10, 17, 18, 23). Another study examining cortisol and state anxiety induced by exam-like stress showed attenuated cortisol response in OC users, but this did not reach statistical significance (37). There might be longer lasting consequences of initiating OCs in early life, i.e., in adolescence when the brain is not fully matured. A recent study found no overall difference between OC users and non-users when exposed to a social stress test, but notably found a reduced response in OC users who had initiated OC prior to puberty relative to in adulthood (20). Also, Bouma et al., 2009, only found a slightly blunted morning cortisol in adolescent females who presumably had used OCs for a short period. Altogether most studies suggest a blunted cortisol response in OC users, which aligns with our finding. This supports that OC use compromises cortisol responsiveness across a range of stimuli of the HPA-axis.

Our secondary analyses were less conclusive: (1) We did not find an estradiol-BY-OC user group effect on CAR and plasma estradiol, and plasma estradiol was not associated with CAR within the OC user group. We speculate this finding may reflect that long-term suppression of sex steroids in OC users is affecting HPA dynamics, in contrast to natural dynamics of estradiol in non-users, independent on the actual levels of estradiol. The estradiol levels in the non-user group were lower than might be expected for natural cycling women, which is most likely because many were recruited in the early follicular phase (26 out of 53 non-user women), which was a requirement in certain studies some of the participants were recruited for. Kirschbaum et al. (1999) only found a CAR difference between oral contraceptive women and normal cycling women in luteal phase but not the follicular phase (17). Thus, this points to a difference across all phases, and one might speculate if our results had been even more evident if we had only included women from the luteal phase. However, as the estradiol sample size was small, these results should be interpreted with caution. (2) As an index for absolute cortisol levels, we examined wake-up cortisol values, which were significantly higher in OC users, and evening cortisol, which did not differ between OC users and non-users. However, there is currently no consensus in terms of absolute levels as both amplified, reduced, and unaltered levels have all been reported (11–13, 36, 38). Interestingly, even after adjusting our main analysis for cortisol awakening values, we continued to see a trend (p=0.058) towards a blunted CAR in OC users. Awakening time (see supplementary) and sleep quality did not affect the association between CAR and OC use. Thus, several properties (circadian and superimposition) of the HPA-axis may well be affected by OC use. As the HPA axis contributes with generating diurnal rhythm, a high wake-up value may also reflect disturbed HPA axis dynamics. Also, it is presumably difficult to generate a forceful CAR if the wake-up value is already high. Absolute evening values did not differ between OC users and non-users. We believe this supports that OC use disturbs the dynamics rather than the absolute cortisol levels per se.

Central mechanism may be involved in the association between OC use and CAR as sex steroid receptors are pervasively expressed in key parts of the neural circuitry controlling the HPA axis, amongst these hippocampus, allowing sex steroids to modify the neuroendocrine response to stress (15). Other factors may also play a role such as changes in the levels of cortisol binding globulin in OC users (13, 16), which ostensibly would decrease the free fraction of cortisol that pass to saliva. However, this does not align with our observation of increased cortisol levels at awakening in OC users.

CAR has been suggested to be a potential marker of hippocampal function where the magnitude of CAR is positively related to hippocampal volume (39), as a well-functioning hippocampus may be necessary for CAR to occur (39). Further, the hippocampus expresses high levels of estrogen receptors and shows great plasticity, which covaries with estrogen (40). Thus, the OC-induced attenuation of CAR could potentially be mediated by a hippocampal pathway. Along this line, Clow et al. (2010) (41) suggested that the hippocampus may play a role in the regulation of CAR prior to awakening. During sleep, the hippocampus inhibits cortisol secretion, and it is particularly active during REM sleep, which is dominant in later stages of sleeping and immediately pre-awakening. At awakening, hippocampal activation switches off (41). This might add to explain the increased awakening cortisol levels and impaired quality of sleep we observed in OC users. The hippocampus also appears to play an important role in the development of depression (42), and hippocampal volume has been linked to OC use (43). Future studies may illuminate if hippocampus volumes are related to OC use in a manner coupled to HPA-axis outputs and dynamics or cognitive performance.

In summary, our findings are in line with the notion that OC-induced suppression of endogenous sex steroids can compromise HPA axis dynamics as demonstrated by a blunted CAR and a change in diurnal features, i.e., cortisol levels at awakening. The effect of initiating OCs at different brain maturation states, especially in adolescence, and the reversibility after cessation of OCs should be investigated in future studies.

### OC Use, Estradiol, and Working Memory

Existing literature consistently demonstrates that sex steroids, in particular estradiol, affect WM in natural cycling, pregnant, and menopausal women (24, 26, 27). Contrary to our expectation, we did not find a significant association between OC use and WM. Also, we did not find an estradiol-by-OC use interaction effect on WM, and the plasma estradiol concentration was not associated with WM within the OC user group. However, a non-significant trend towards better SDMT performance in OC users was observed, but this might be a spurious finding. Notably, previous studies reporting an association between sex steroids and WM function used visuo-spatial WM tasks including spatial WM, paragraph recall, and mental rotation (24, 26, 27). This could help explain why we observed a trend-like improvement in performance for OC users on the visual SDMT task but not the verbal LNS task. It should also be noted that the SDMT task does not exclusively assess WM functioning, but it is also commonly used to index processing speed (44); the observed trend could therefore reflect changes in processing speed instead of WM function. In addition, the LNS task design makes it particularly challenging and mentally taxing for the participant. Decreased perseverance in hormonal contraceptive users has been linked to worse performance on both simple and challenging tasks (45), and we speculate if this may be related to a less sensitive HPA axis, which would lower the capacity for OC users to mobilize extra cognitive resources. We also speculate if the combination of reduced perseverance and the more challenging auditive nature of the LNS task may explain why we did not see a similar trend with LNS as with the less challenging SDMT. In summary, we find no evidence for impaired WM in OC users, and the trend-like effect observed might be spurious. However, our sample size was modest with regard to detecting moderate to smaller effects on cognition, and we cannot exclude that such effects could be detected in larger sample sizes. Furthermore, effects on different cognitive domains need to be disentangled.

### OC Use and Risk for Depression

Our results suggest that OC users are not able to mobilize an efficient HPA axis response to a daily stimulus as the transition from sleep to awake. This may also imply that OC users have a limited biological capacity to handle stress, which can add as a risk factor for mood disorders. Interestingly, we also observed that OC users scored higher on mood (TMD) and displayed worse sleep relative to non-users, perhaps suggesting subclinical effects correlate to a less dynamic HPA axis function in OC users. Frokjaer et al. (2015) demonstrated that gonadotropin-releasing hormone-agonist (GnRHa)-induced suppression of endogenous sex steroids in healthy women triggers subclinical depressive symptoms (46). This points to possible subclinical effects from ovarian sex steroid suppression in otherwise healthy individuals. Several large epidemiological studies have suggested an association between OCs and depression, especially among adolescents (3, 4, 6). It is also important to emphasize that the majority of women using OCs tolerate them well and do not experience adverse effects. However, a particular subgroup of vulnerable or hormone-sensitive individuals could be at risk of developing depressive episodes when exposed to hormonal transitions. Potential estrogen-sensitive transcripts predicting hormone-induced mood changes have been identified; however, it is unknown if these markers of estrogen sensitivity translates to OC use or other, naturally occurring, hormonal transitions (47). Future work should consider intervention studies to illuminate potential causal links between use of hormonal contraceptives, changes in HPA axis dynamics and potential emergence of depressive symptoms.

### Methodological Considerations and Limitations

Our study has important strengths and limitations that should be considered when interpreting our findings. A main strength is that we have detailed CAR measures in a larger sample compared to earlier studies (Kirschbaum et al., 1995: n=59; and Kirschbaum et al., 1999: n=61) and adjusted for relevant covariates. Furthermore, our sample comprised healthy women exposed to an everyday HPA axis stimulus, i.e., the natural transition from sleep to awakening. This allowed us to investigate HPA-axis dynamics in the absence of psychosocial stress. As a limitation, first, a possible “healthy user” bias might exist in our sample since (a) the women included in our study did not develop depression after starting OCs, and (b) they did not experience any mood deteriorations extensive enough to terminate the use of OCs. Therefore, we cannot exclude that the association between OC use and CAR might be stronger in high-risk or patient groups. Second, the cortisol measures in our study were determined by two different methods due to changes in the hospital laboratory across the data collection period. Although we observed no batch effects and the frequency of data points determined with the two methods were equally distributed between OC user and no-user groups, this may have added larger variation in our data, which in terms may have reduced our power to detect a difference between groups. Third, there were some limitations regarding menstrual cycle phase data and estradiol: (a) Information on menstrual phase were not available for all naturally cycling women in the non-user group, which limits our ability to control for menstrual phase effects. (b) Ideally, plasma estradiol should have been collected the same day as the cortisol saliva samples and not up to 3 days later. (c) The analyses with estradiol had small sample sizes. (d) Time of day for the estradiol measure was not standardized. Since subtle diurnal fluctuations cannot be excluded, one may speculate if it has added variation to our data.

Fourth, we did not have data on which kind of oral contraceptive was used by the women nor if the women collected their cortisol samples on an active or an inactive pill day. Finally, causality cannot be inferred because of the cross-sectional nature of the study.

Consistent with our hypothesis, we observed that women who use oral contraceptives have blunted cortisol dynamics relative to non-users. OC use did not appear to be coupled to WM performance relative to non-users; however, a trend towards better WM performance on the SDMT was observed in OC users. We speculate that the observed effects on the HPA axis may lead to an inadequate response to stress that in an adverse environment may contribute as a risk factor for depression in sensitive individuals.

## Data Availability Statement

Due to the General Data Protection Regulation, the data that support the findings of this study are not readily available. Data in the Cimbi database can be accessed by application (http://www.cimbi.dk/db).

## Ethics Statement

The studies involving human participants were reviewed and approved by the Capital Region of Denmark, Ethical Committee. The patients/participants provided their written informed consent to participate in this study.

## Author Contributions

EH: Data curation, formal analysis, investigation, methodology, project administration, software, visualization, validation, writing—original draft. CB: Validation, writing—review and editing. VD: writing—review and editing. AN: Resources, writing—review and editing; NJ: Resources, writing—review and editing; BO: Formal analysis, software, data curation, supervision, writing—review and editing. DS: Supervision, writing—review and editing. VF: Conceptualization, funding acquisition, resources, investigation, methodology, validation, writing—original draft, supervision. All authors contributed to the article and approved the submitted version.

## Funding

This work was supported by the Independent Research Fund, Denmark [grant number: 7025-00111B]; Lundbeckfonden, Denmark [No grant number] and Desirée and Niels Yde Foundation [grant number: 481-17].

## Conflict of Interest

The authors declare that the research was conducted in the absence of any commercial or financial relationships that could be construed as a potential conflict of interest.

## Publisher’s Note

All claims expressed in this article are solely those of the authors and do not necessarily represent those of their affiliated organizations, or those of the publisher, the editors and the reviewers. Any product that may be evaluated in this article, or claim that may be made by its manufacturer, is not guaranteed or endorsed by the publisher.

## Supplementary Material

Supplementary Analyses 1 Main analysis including hormonal IUD users in non-user group. When including hormonal IUD users in the non-user group, we found similar results as in our main analysis.

Supplementary Analyses 2 Main analysis adjusting for awakening time instead of work day status. Replacing work day status with awakening time did not change our results as awakening time did not contribute to the model (p-value = 0.79).

Supplementary Figure 6 Selection of participants for estradiol interaction analysis.

Supplementary Figure 7 Selection of participants for working memory analysis.

Supplementary Table 4 CAR regression report for (A) Main model, (B) Model including all covariates in CAR analysis.

Supplementary Table 5 Regression report for main WM analyses including potential covariates: **(A)** LNS, **(B)** SDMT. **(A)** Analysis includes data from 64 women as information on PSQI was not available on all women. **(B)** Analysis includes data from 62 women as information on PSQI was not available on all women.

## Abbreviations

OCs, oral contraceptives; HPG, hypothalamic-pituitary-gonadal; HPA, hypothalamic-pituitary-adrenal; CAR, cortisol awakening response; WM, working memory; IUD, intrauterine devices**;** AUCi, area under the curve with respect to increase from baseline at awakening; LNS, letter number sequencing; SDMT, symbol digit modalities test.
