# Supplementary material for: Stress-Hormone Dynamics and Working Memory in Healthy Women Who Use Oral Contraceptives Versus Non-Users
Source: Front Endocrinol (Lausanne). 2021 Nov 8;12:731994. doi: 10.3389/fendo.2021.731994 (PMC8606688; doi:10.3389/fendo.2021.731994)
Supplement: Supplementary Analyses 1 — Main analysis including hormonal IUD users in non-user group. When including hormonal IUD users in the non-user group, we found similar results as in our main analysis. [file Table_1.pdf]

1. **Main analysis including hormonal IUD users in non-user group**

**β**	**CI 95 %**	**p-value**
-181	[-315:-46]	0.01

When including hormonal IUD users in the non-user group, we found similar results as in our main analysis.
