# Supplementary material for: Stress-Hormone Dynamics and Working Memory in Healthy Women Who Use Oral Contraceptives Versus Non-Users
Source: Front Endocrinol (Lausanne). 2021 Nov 8;12:731994. doi: 10.3389/fendo.2021.731994 (PMC8606688; doi:10.3389/fendo.2021.731994)
Supplement: Supplementary Analyses 2 — Main analysis adjusting for awakening time instead of work day status. Replacing work day status with awakening time did not change our results as awakening time did not contribute to the model (p-value = 0.79). [file Table_2.pdf]

2. **Main analysis adjusting for awakening time instead of work day status**

**β**	**CI 95 %**	**p-value**
-200	[-327;-58]	0.005

Replacing work day status with awakening time did not change our results as awakening time did not contribute to the model (p-value = 0.79).
