# Supplementary figures and images for: Stress-Hormone Dynamics and Working Memory in Healthy Women Who Use Oral Contraceptives Versus Non-Users
Source: Front Endocrinol (Lausanne). 2021 Nov 8;12:731994. doi: 10.3389/fendo.2021.731994 (PMC8606688; doi:10.3389/fendo.2021.731994)

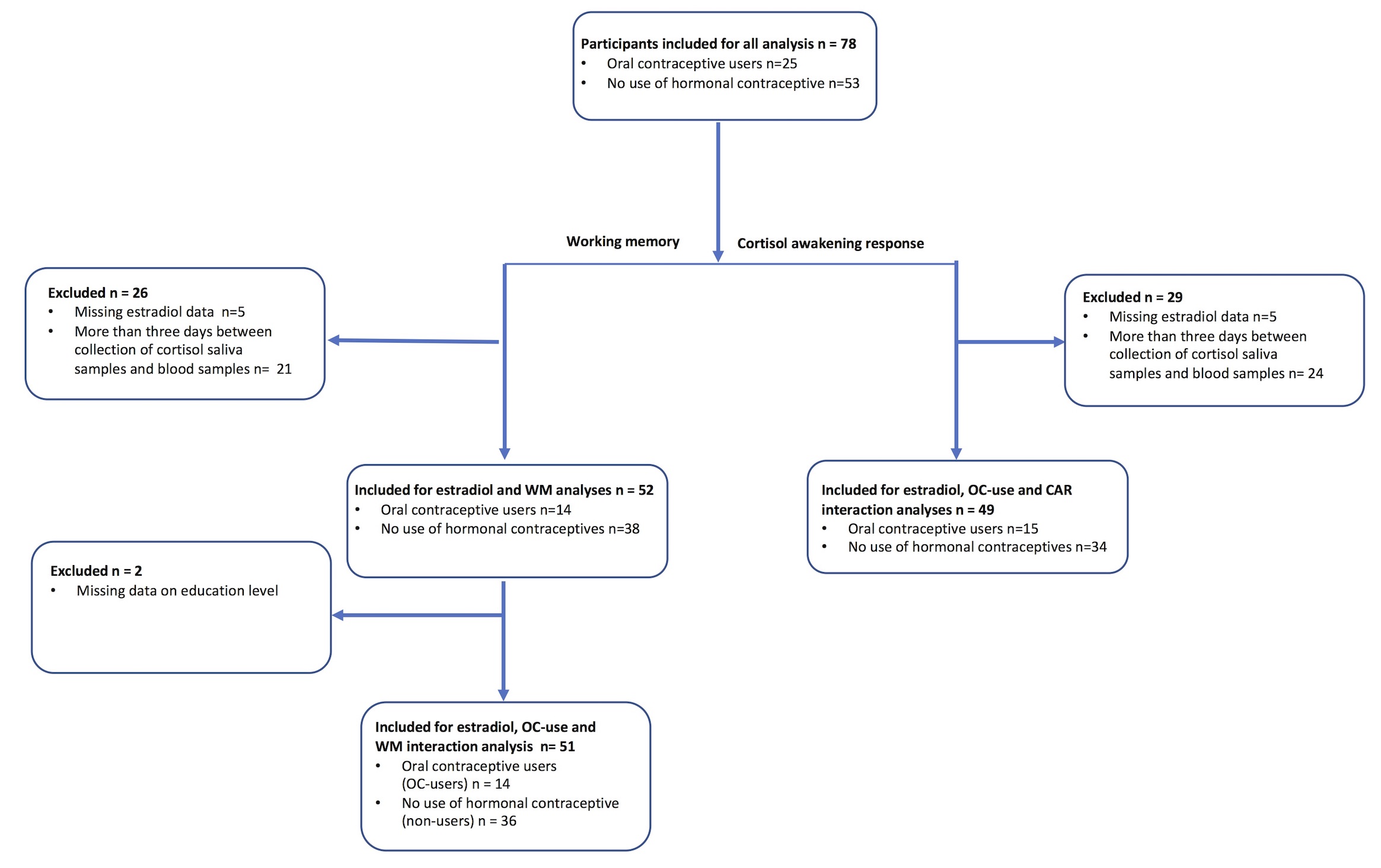

Supplement: Supplementary Figure 6 — Selection of participants for estradiol interaction analysis. [file Image_1.jpg]

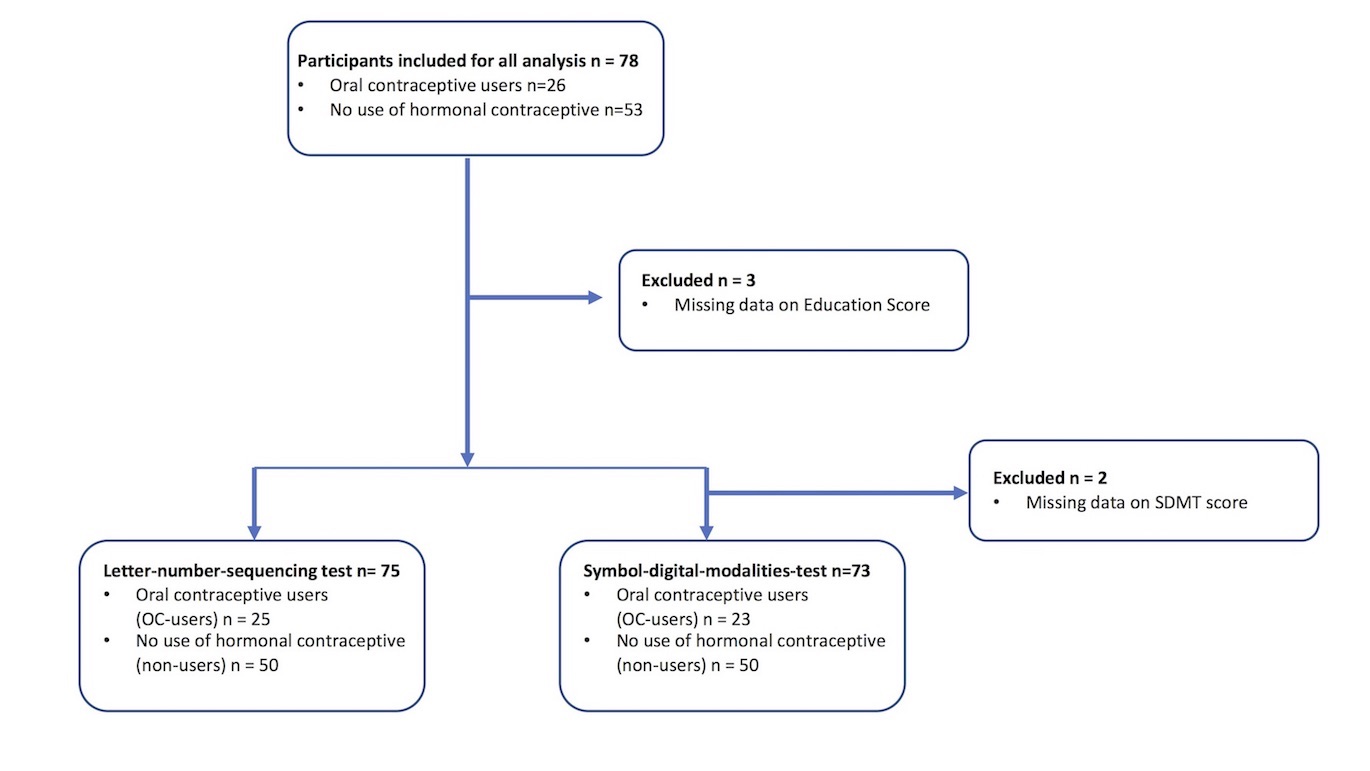

Supplement: Supplementary Figure 7 — Selection of participants for working memory analysis. [file Image_2.jpg]
