# Supplementary material for: Stress-Hormone Dynamics and Working Memory in Healthy Women Who Use Oral Contraceptives Versus Non-Users
Source: Front Endocrinol (Lausanne). 2021 Nov 8;12:731994. doi: 10.3389/fendo.2021.731994 (PMC8606688; doi:10.3389/fendo.2021.731994)
Supplement: Supplementary Table 4 — CAR regression report for (A) Main model, (B) Model including all covariates in CAR analysis. [file Table_4.pdf]

Table 4: Regression report for main WM analyses including potential covariates: A. LNS, B. SDMT

A.

Coefficients	Value / Effect size	Standard Error	P-value
LNS	0.16	0.89	0.85
Education score	-0.04	0.30	0.90
Age	-0.03	0.08	0.68
BMI	-0.14	0.16	0.39
Smoking status	-0.07	1.34	0.96
TMD	0.02	0.03	0.39
PSQI	-0.30	0.17	0.17

Analysis includes data 64 women as information on PSQI was not available on all women.

B.

Coefficients	Value / Effect size	Standard Error	P-value
SDMT	4.2	3.09	0.18
Education score	1.5	0.92	0.10
Age	0.16	0.23	0.49
BMI	0.43	0.49	0.37
Smoking status	4.58	4.07	0.26
TMD	-0.08	0.09	0.38
PSQI	0.46	0.56	0.41

Analysis includes data 62 women as information on PSQI was not available on all women.
