# Supplementary material for: Stress-Hormone Dynamics and Working Memory in Healthy Women Who Use Oral Contraceptives Versus Non-Users
Source: Front Endocrinol (Lausanne). 2021 Nov 8;12:731994. doi: 10.3389/fendo.2021.731994 (PMC8606688; doi:10.3389/fendo.2021.731994)
Supplement: Supplementary Table 5 — Regression report for main WM analyses including potential covariates: (A) LNS, (B) SDMT. (A) Analysis includes data from 64 women as information on PSQI was not available on all women. (B) Analysis includes data from 62 women as information on PSQI was not available on all women. [file Table_3.pdf]

Table 3: CAR regression report for A. Main model, B. Model including all covariates in CAR analysis

A.

Coefficients	Value / Effect size	Standard Error	P-value
OC-use	-203	367.5	0.006
Age	-9.7	8.404	0.25
Workday status	-0.17	67.30	0.99
BMI	23.69	14.66	0.11

B.

Coefficients	Value / Effect size	Standard Error	P-value
OC-use (OC-user)	-238	92.4	0.013
Age	11.2	10.0	0.27
Work day	-2.56	81.71	0.98
BMI	20.9	18.36	0.26
PSQI	17.6	16.77	0.30
5HTLLPR genotype	-6.97	90.24	0.94
Cohens PSS	-5.86	7.86	0.46
TMD	0.69	3.44	0.84
Smoking (No smoker)	101	145.1	0.49
Season	26.6	86.76	0.76
